# Evaluation of Cognitive levels and Item writing flaws in Medical Pharmacology Internal Assessment Examinations

**DOI:** 10.12669/pjms.334.12887

**Published:** 2017

**Authors:** Saba Tariq, Sundus Tariq, Sadia Maqsood, Shireen Jawed, Mukhtiar Baig

**Affiliations:** 1 Dr. Saba Tariq, MBBS, M.Phil, Assistant Professor, Pharmacology, University Medical & Dental College, Faisalabad, Pakistan; 2 Dr. Sundus Tariq, MBBS, M.Phil Assistant Professor, Physiology, University Medical & Dental College, Faisalabad, Pakistan; 3 Dr. Sadia Maqsood, MBBS, M.Phil Senior Demonstrator, Pharmacology, Shaikh Zayed Postgraduate Medical Institute, Shaikh Zayed Hospital, Lahore, Pakistan; 4 Dr. Shireen Jawed, MBBS, M.Phil Assistant Professor, Physiology, Aziz Fatima Medical College, Faisalabad, Pakistan; 5 Dr. Mukhtiar Baig, MBBS, M.Phil, PhD Professor of Clinical Biochemistry, Faculty of Medicine, Rabigh, King Abdulaziz University, Jeddah, KSA

**Keywords:** Assessment, Item analysis, MCQ

## Abstract

**Objectives:**

This study aimed to evaluate the cognitive levels of Multiple Choice Questions (MCQs) & Short Answer Questions (SAQs) and types of Item Writing Flaws (IWFs) in MCQs in Medical Pharmacology internal assessment exams.

**Methods:**

This descriptive, study was conducted over a period of six months, from December 2015 to May 2016 and evaluated six internal assessment examinations comprising SAQs and MCQs. A total of 150 MCQs and 43 SAQs were analyzed. These questions were administered to third-year medical students in the year of 2015. All SAQs were reviewed for their cognitive levels and MCQs were reviewed for cognitive levels as well as for IWFs. Items were classified as flawed if they contained one or more than one flaw. The cognitive level of the questions was determined by the modified Bloom’s taxonomy.

**Results:**

The proportion of flawed items out of 150 items in six exams ranged from 16% to 52%. While the percentage of total flawed items was 28%. Most common types of flaws were implausible distractors 19.69% (26), extra detail in correct option 18.18% (24), vague terms 9.85% (13), unfocused stem 9.09% (12) and absolute terms 9.09% (12). The two-third of MCQs 97(64.67%) were assessing the recall of information, while 29 (19.33%) and 24 (16%) were assessing the interpretation of data and problem-solving skills respectively. The majority of the SAQs (90.7%) were assessing recall of the information and only 9.3% were assessing interpretation of data while none of the questions was assessing the problem-solving skills.

**Conclusions:**

The cognitive level of assessment tools (SAQs & MCQs) is low, and IWFS are common in the MCQs. Therefore, faculty should be urged and groomed to design problem-solving questions which are devoid of any flaws.

## INTRODUCTION

The assessment either summative or formative has a powerful effect on learning and is considered an essential variable in leading the learners for achieving the goal.^1^ The assessment of knowledge and competence of undergraduate medical students has immense importance because they need to be a safe practitioner in future.^2^

From assessment point of view, it is always important that assessment should be reliable and valid and be able to differentiate between satisfactory and unsatisfactory performers.^3^ The way of assessment influences the students’ choice of learning approach.^4^,^5^ If the quality of the questions are not up to the mark and the majority of the questions are just testing the recall of the isolated facts then such assessment would promote the superficial learning approach.^6^

There are several types of written tests like long essay questions, short answer question, modified essay questions, MCQs, extended matching MCQs, etc. It is mentioned in the literature that essay type questions can be used in the recall of knowledge and higher order cognitive levels questions both.^7^ Moreover, in undergraduate setting essay type questions are more suitable.^8^

In Pakistan, Pakistan Medical and Dental Council (PMDC) and Higher Education Commission (HEC) have recommended multiple formats of assessment. Thus all the affiliated colleges of University of Health Sciences (UHS), Lahore, are using various formats like MCQs, SAQs, OSPE/OSCE and Viva. It is also recommended in medical education that medical students should be assessed by various assessment tools.^9^

MCQs are frequently used because of objectivity, elimination of assessor’s favoritism and extensive coverage of the subject in a short period.^10^ As compared to MCQs, the marking of SAQs is time-consuming, expensive, and may involve assessor’s biases.

Item-writing flaws (IWFs) arise when we deviate from the accepted guidelines of making MCQs, and consequently, such MCQs affect the performance of the students in such a way that it might become difficult or easier for the student to answer it.^11^ A study reported that more than 90% of MCQs in an examination were of low cognitive levels and that 46.2% of these MCQs had item writing flaws in them.^12^ The authors also observed that MCQs constructed at lower cognitive levels have more item writing flaws. A study in Pakistan reported that the cognitive level of most of the SEQs (83.33%) and MCQs (60%) were at recall level, respectively, and 69 IWFs (46%) were found in 150 MCQs.^6^

The designing of a high-quality MCQs is a skill, and like other skills, it needs training and practice without that there is more probability that MCQs would have more IWFs.

Our College has faculty with a diverse background, and few are trained and well experienced, while others are new in teaching. So we assume that cognitive level of our written assessment is not up to the mark, and it is likely that our MCQs have several IWFs. Until now in our college, no such study was done to evaluate written assessment tools.

Therefore, this study aimed to evaluate the cognitive levels of MCQs & SAQs and types of item writing flaws in MCQs in six Pharmacology and Therapeutics internal assessment exams held in 2015.

## METHODS

The descriptive study was conducted on six internal assessment examination comprising SAQs and MCQs from Pharmacology & Therapeutics Department, the University Medical and Dental College, Faisalabad, Pakistan in the year 2015.

In each internal assessment exam, there were 25 MCQ and 5- 10 SAQs. A total of 150 MCQs and 43 SAQs from six internal examinations of 2015 were evaluated for their cognitive levels and IWFs. For identifying types of IWF’s standard criteria given by several educationists were used and fourteen (14) frequently occurring violations of item-writing guidelines were selected from literature & were subsequently applied to assess the quality of the 150 MCQs in all six exams.^12^-^14^

A subject expert and a medical educationist’s were taken in the team from outside the University for analyzing the questions’ cognitive levels and IWFs. A proforma was prepared to evaluate each MCQ, and SAQ and each reviewer assessed the questions individually by the predefined criteria and difference of opinion between the medical educationist, and subject experts were further sorted out for reaching an agreement about the controversial questions.

The questions’ cognitive levels were evaluated by the Buckwalter’s modification^15^ of the Bloom’s taxonomy.^16^ Buckwalter et al., (1981) modified Blooms six cognitive domains into three levels.^15^ Level I: Incorporate questions that try to test recall of information. Level II: Incorporate questions that try to check understanding and interpretation of data. Level III: Incorporate questions that try to check the application of knowledge for resolving a peculiar problem.

Items were classified as flawed if they contained one or more than one flaw. The ethical review committee of the College gave the approval for this study.

### Data analysis

The SPSS version 21 was utilized to analyze the data and frequencies and percentages were calculated.

## RESULTS

**Fig. 1:**
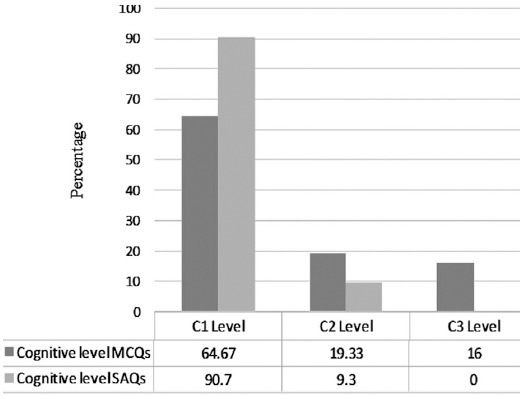
Cognitive level of MCQs and SAQs in all six exams

The descriptive statistics for each internal assessment exam is given in Table-I. The proportion of flawed items in six exams ranged from 16% to 52%, and the percentage of total flawed items out of 150 was 28%. Most common types of flaws were implausible distractors 19.69% (26), extra detail in correct option 18.18% (24), vague terms 9.85% (13), unfocused stem 9.09% (12) and absolute terms 9.09% (12) (Table-II). Two-third of MCQs 97(64.67%) were assessing the recall of information, while 29 (19.33%) and 24 (16%) were assessing the interpretation of data and problem solving respectively (Fig. 1). Neither of the SAQs was assessing the problem-solving skills, and only 9.3% were assessing the interpretation of data while remaining 90.7% were assessing recall of information (Fig. 1).

**Table-I:** Descriptive Statistics on Tests.

	*T1*	*T2*	*T3*	*T4*	*T5*	*T6*	*Total*
No of Items	25	25	25	25	25	25	150
No of flawed items	13	11	9	7	5	4	43
% of flawed items	52	44	36	28	20	16	28

**Table-II:** Types of item writing flaws in six tests.

*Types of IWF*	*T1*	*T2*	*T3*	*T4*	*T5*	*T6*	*Total*
Absolute terms	3	3	2	2	2	0	12
Vague terms	4	3	3	1	1	1	13
Implausible distractors	6	6	4	3	5	2	26
Extra details in correct option	6	5	5	4	2	2	24
Negative stem	2	1	0	0	0	0	3
Grammatical clues	2	1	0	1	0	0	4
Logical clues	2	1	0	1	1	0	5
Word repeats	1	2	2	1	1	1	8
> 1 correct answer	2	1	2	0	0	0	5
Unnecessary information in stem	2	2	1	0	1	0	6
Lost sequences in data	3	3	2	1	1	0	10
All of the above	2	1	0	0	0	0	3
None of the above	1	0	0	0	0	0	1
Unfocused stems	3	3	2	1	2	1	12
Total	40	33	24	14	14	7	132
Fail to cover the option test	10	8	8	6	5	3	40

* Total number of flaws in 06 exams is not equal to the total number of flawed items as some questions had more than one flaw.

## DISCUSSION

The current study evaluated the quality of six internal assessment examinations of 3^rd^ year MBBS students. These exams comprised of 150 MCQs and 43 SAQs. Analysis for cognitive levels based on Bloom’s criteria showed that of 60.47% of MCQS were at low cognitive levels. These results are similar to another research that pointed out 46.2 % IWFs in MCQS for nursing assessment, and 90% of the questions were of the low cognitive level.^17^ Downing (2002) had reported that 33-46% of MCQS had violations of item writing guidelines.^18^

Our results are similar to a study that found the cognitive level of the majority of SEQs (83.33%) was at recall level while very less number of questions (16.67%) were evaluating interpretation of data.^6^ Our MCQs analysis results are dissimilar to the same study regarding recall questions and problem-solving questions that study reported 76% recall questions and 0% problem-solving MCQs while we had 64% recall questions and 16% problem-solving MCQ in our exam.

Quantitative analysis of our data indicates that there were 43 IWFs (28%) in 150 MCQs while another study in Pakistan reported 69 IWFs (46%) in 150 MCQs.^6^ In the present study, we noted an important observation that the number of flawed items decreased from exam one to six. This shows the gradual improvement in the IWFs of the items, and it could be due to the teachers training course arranged by the Department of Medical Education in the University, and few of faculty members of Pharmacology and Therapeutics Department have attended that course. Therefore, there were fewer IWFs and the higher percentage of interpretation and problem-solving questions.

The types of IWFs found in our study (the commonest among them were implausible distractors, unfocused stem and unnecessary information in the stem) are similar to numerous other studies.^3^,^6^,^12^,^19^,^20^

Additionally, we also noticed that sixth assessment exam of our evaluation had less number of flawed items (16%) and had a greater passing score (70%) as compared to first, second, third and other exams. This finding suggests that flawed item negatively affects the high passing scores which are in agreement with another report.^20^ A study by Karelia et al., (2013) analyzed the MCQs of pharmacology summative tests of medical students and pointed that ambiguous wording, wrong keys, gray areas of opinion, and questions from areas of disagreement were few common causes for the poor discrimination.^21^

A recent study by Patil et al., (2016) reported that only 10% of MCQs fulfilled all the criteria for an ideal MCQ.^1^ A study evaluated Therapeutics MCQs and concluded that exams with well-constructed MCQs that target various cognitive levels could be a valid assessment of students performance.^22^

It is essential to design questions that investigate a deeper understanding of the topic/subject, and that stipulate the application of higher-order thinking skills to incorporate Basic Sciences knowledge with relevant, clinically-oriented contexts.^23^ It is suggested that we should focus on the construction of problem-solving questions rather than the recall of information because problem-solving questions help in long-term retention of knowledge. A study at the University of Health Sciences (UHS) has reported that properly designed MCQs with high construct & context validity can be used not only to assess knowledge but also higher cognitive skills.^24^

Our study suggests that there is a strong requirement to work on the improvement of assessment tools by initiating faculty development program. The selection or construction of a good MCQ is the most critical step in assessing the knowledge of the student and to differentiate students with different capabilities.^25^ The quality of a good MCQ mainly depends on following a standard protocol of making an MCQ; that is the correction of flawed items, replacement of non-functioning distractors and improving the cognitive level.^26^ However, the faculty training in this regard is also very important.^27^

A study by Naeem et al., (2011) at Aga Khan University (AKU) observed that substantial improvement in item quality is dependent on faculty development.^28^ Another study reported 17% change in the quality of MCQs after attending a short training session about the construction of MCQS.^29^ Other methods of assessment are also gaining popularity as they have less chance of flawed items and are also improving student test scores.^30^

### Limitation of the study

The present study has several limitations; firstly, we analyzed the written tests of only one subject in a medical college. Therefore, our results cannot be generalized and don’t t reflect the quality and IWFs present in other subjects and colleges affiliated with UHS. Secondly, we did not calculate the discrimination and difficulty indices.

## CONCLUSION

The cognitive level of assessment tools (SAQs & MCQs) is low, and IWFs are very common in the MCQs.

### Recommendation

A careful review of the learning outcomes is required and how they are aligned with the assessment. A test blueprint should be developed before the exam, and all questions should be constructed according to the cognitive level mentioned in that test blueprint. The faculty should be trained for the construction of MCQs for higher cognitive levels and developing test blueprint. The properly trained faculty may play an important role not only in minimizing item-writing flaws but also improving cognitive levels.

### Author’s Contribution

**Saba Tariq** designed the study, collected the data and drafted the manuscript.

**Sundus Tariq** data entry and analysis, layout design, manuscript writing.

**Sadia Maqsood** data analysis, table’s formulation and manuscript writing.

**Shireen Jawed** contributed in literature review, proof reading and correction

**Mukhtiar Baig** helped in data analysis, drafting, reviewing and editing the final manuscript.
